# Not your Typical Rash: A Case of IgA Nephropathy in the Setting of HIV

**DOI:** 10.7759/cureus.5368

**Published:** 2019-08-12

**Authors:** Pamela Contreras-Chavez, Andrea Anampa-Guzmán, Jose Henao, Raynieri Fernandez, Peguy Saad

**Affiliations:** 1 Internal Medicine, Advocate Illinois Masonic Medical Center, Chicago, USA; 2 Internal Medicine, Main National University of San Marcos, Lima, PER

**Keywords:** aids, henoch-schönlein purpura

## Abstract

Human immunodeficiency virus (HIV) infection presents with a variety of conditions. We describe the case of a 33-year-old Hispanic male with IgA nephropathy and Henoch-Schonlein Purpura in the setting of HIV. The incidence of vasculitis associated with HIV infection is less than 1%. There are few cases reported of IgA nephropathy in the context of HIV. Henoch-Schonlein Purpura usually presents in children. We encountered a patient with rare illnesses while in the setting of immunodeficiency.

## Introduction

Human immunodeficiency virus (HIV) infection presents with a variety of conditions. Leukocytoclastic vasculitis, also called hypersensitivity vasculitis, is the inflammation of small blood vessels. Debris of neutrophils can be seen within the blood vessel walls. We report a case of leukocytoclastic vasculitis in the setting of an HIV/AIDS infected patient.

## Case presentation

A 33-year-old Hispanic male with a past medical history significant for HIV/AIDS non-compliant with medical treatment presented to the emergency department (ED) complaining of worsening lower extremity petechial rash and edema. The rash was initially described as non-blanching, non-pruritic erythema limited to both ankles accompanied by a burning sensation in the sole of the feet that started four weeks ago. This was the first time he experienced such a rash. The patient was sexually active with multiple partners, both men, and women. He had a recent trip to Mexico, with no sick contacts. His last CD4 count (one month ago) was 74, and viral load around 1 million.

The patient went to another hospital when the symptoms began. There he was started on treatment for secondary syphilis with intramuscular (IM) penicillin since he had positive rapid plasma reagin (RPR) with a titer of 1:256. He was discharged from the hospital one day later, and advised to get started on antiretroviral therapy and prophylaxis with Trimethoprim /Sulfamethoxazole (Bactrim), and Azithromycin with close follow up. Two weeks after the initial presentation, he went to his primary care physician due to unresolved and worsening symptoms, including bilateral lower extremity edema and ascending rash that was present on the lower back, and abdomen with associated poly-arthralgias in the hands, ankles, and knees. 

He was advised to return to the ED for further care and synovial fluid analysis which he refused. His presentation and physical exam findings were thought to be secondary to disseminated gonococcal infection versus possible sulfa related allergy. The decision was made to send for Gonococcal/Chlamydia urine test and to give empiric antibiotic treatment with Ceftriaxone. His prophylaxis was also switched from Bactrim to Dapsone. Despite this treatment, his clinical condition deteriorated, associated with generalized fatigue and palpitations. The patient decided to go to the ED because he was unable to ambulate due to generalized lower extremity edema.

On arrival, vital signs were significant for tachycardia. Physical examination was remarkable for a diffuse non-blanching erythematous/purpuric rash on the flexor and extensor surface of both upper and lower extremities, as well as the anterior aspect of the chest, abdomen, and lower back. Lymphadenopathy was not found, and there was a full range of motion of all joints with mild swelling in hands and no pain associated with the feet. Initial laboratory studies were notable for a normal complete blood count, basic metabolic, and liver function panel. Urine analysis was remarkable for hematuria (occult blood moderate, erythrocytes 6-10) and proteinuria (protein >500 mg/dL). The decision was made to admit the patient for further care.

As part of the initial management, intravenous saline was given. Rickettsia panel was ordered, and antimicrobials changed to doxycycline. Infectious disease, Nephrology, and Dermatology services were consulted. The recommendations at the time were to obtain extensive laboratory workup that was remarkable for elevated protein/creatinine ratio (1,669 mg/gm), elevated IgA levels (648 mg/dL), repeated RPR positive (1:64) which showed adequate response for treatment given one month ago, elevated erythrocyte sedimentation rate (55 mm/hr), and c-reactive protein (2.5 mg/dL). The repeat CD4 count was 172, and complement levels were within normal limits. The rickettsia panel, hepatitis panel, comprehensive immunologic panel (including antinuclear antibodies with reflex, cytoplasmic neutrophilic antibody, perinuclear neutrophilic antibody) were negative. According to the Infectious Disease team, Doxycycline was discontinued. Anti-retroviral management and Dapsone were resumed. Kidney and skin biopsy were done to search for the etiology of the patient's proteinuria and purpuric skin lesions.

At this point the etiology for the patient's kidney disease was unclear. The differential diagnosis included syphilis which usually causes membranous nephropathy, and HIV nephropathy. HIV nephropathy was less likely since it often presents as a rapidly progressive disease that occurs mostly in African-Americans. Since the patient had elevated IgA and due to his initial clinical presentation with rash, arthralgia, and proteinuria, Henoch-Schönlein purpura was considered in the differential diagnosis. 

**Figure 1:**
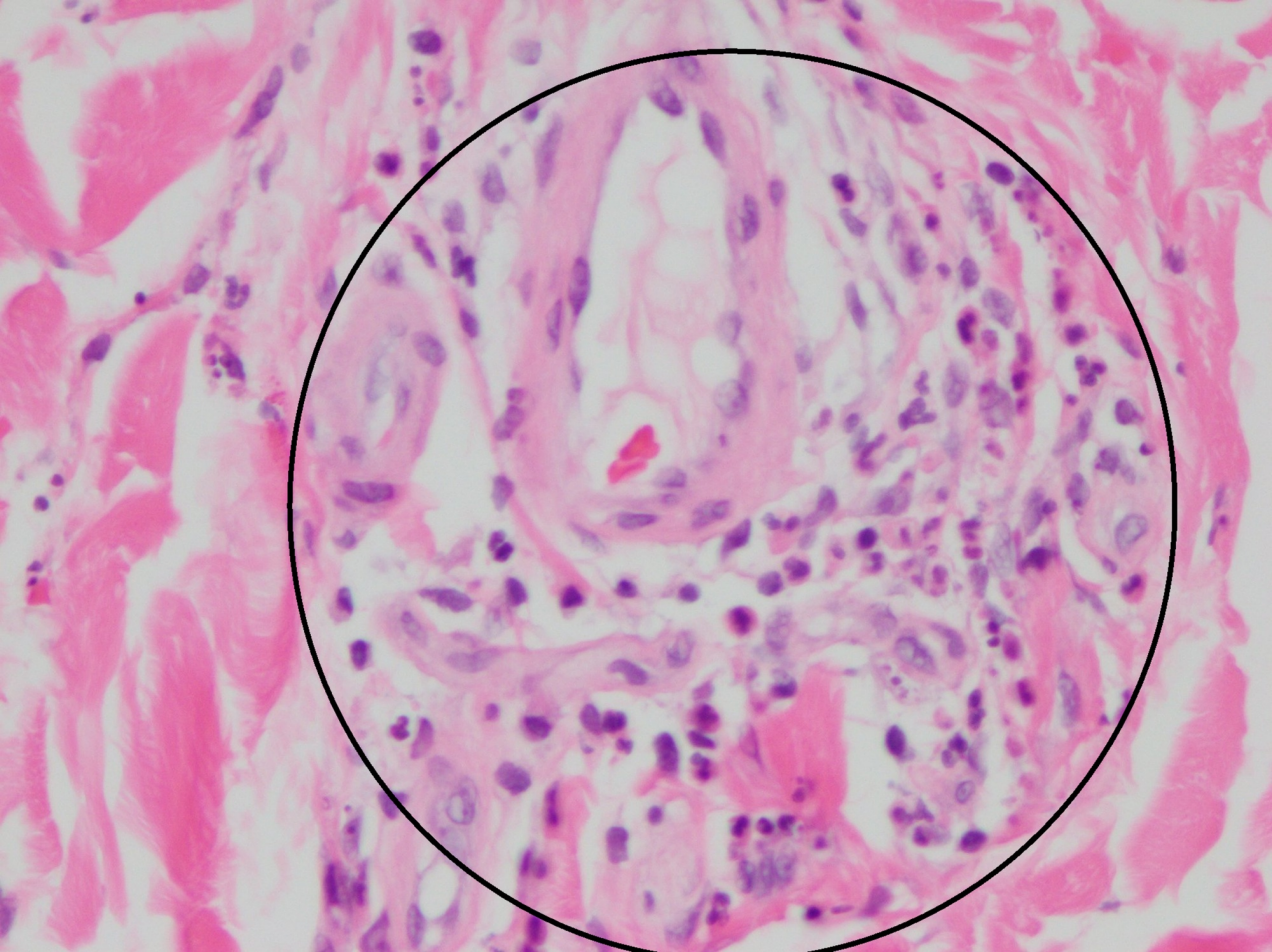
Skin biopsy Perivascular neutrophils and neutrophilic nuclear debris inside the circle (the leukocytoclastic part of leukocytoclastic vasculitis)

**Figure 2:**
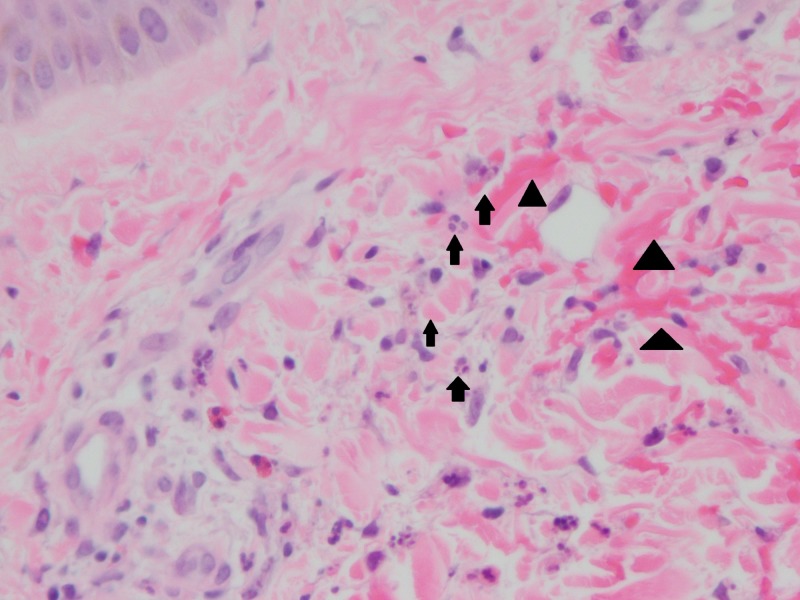
Skin biopsy Neutrophils and neutrophilic nuclear debris are marked with arrows. Extravasated red blood cell in the superficial dermis are marked with arrowheads

The kidney biopsy showed IgA nephropathy with cellular crescents and evolving acute tubular injury (Figure 1 and 2), and the skin biopsy of the left lower extremity, on the other hand, demonstrated perivascular neutrophilic inflammation with karyorrhexis and red blood cell extravasation, suspicious for leukocytoclastic vasculitis. Per rheumatology recommendations, the patient was started on a course of steroids with prednisone (0.5mg/kg four times a day for two weeks) and colchicine (0.6 mg/kg daily) with complete resolution of the symptoms.

## Discussion

We would like to highlight the interesting clinical correlation in our patient with HIV and Henoch-Schönlein purpura (HSP) based on the fact that HIV can be associated with vasculitis, but not commonly with HSP especially in adults. We will focus to describe HSP and IgA nephropathy with HIV. We will describe IgA nephropathy primarily caused by HIV. Even though, this presentation has been reported before, it is not very common [1]. 

Within the rubric of hypersensitivity vasculitis are several vasculitides that manifest in the skin and display a small leukocytoclastic vessel involvement. HSP (Henoch-Schonlein Purpura), drug-induced hypersensitivity vasculitis and cryoglobulinemia have all been encountered in the HIV setting. The incidence of vasculitis associated with HIV infection is less than 1% [2]. HSP is an acute Ig-A mediated disorder characterized by the involvement of small-sized vessels with multiple organ involvement. The pediatric form is more frequent than the adult form showing an incidence of 22 per 100,000 person-years compared to four per 100,000 person-years [3]. Others mention a frequency of 33 times more common in children than adults, with very few case reports of adult-focused disease [4].

HSP in adults can reveal or be associated with HIV; however, few cases have been reported. The pathogenesis of HSP in patients with HIV infection remains hypothetic. Increase of immune complexes circulating in the blood associated with the HIV infection or local replication of HIV itself has been suspected but never demonstrated. HIV infection causes polyclonal hypergammaglobulinemia with a particular elevation of IgG and IgA [4]. Hypergammaglobulinemia is hypothesized to result from chronic antigenic stimulation from HIV virus leading to B-cell hyperplasia [5]. On the other hand, HIV associated nephropathy (HIVAN) is well known and is usually accompanied by heavy proteinuria and occurs almost exclusively in patients of African descent. The most common renal compromise with HIV is focal segmental glomerulosclerosis (FSGS) [6].

Development of polyclonal hypergammaglobulinemia from HIV, and circulating immune complexes frequently are found [7]. Kimmel et al. showed that immune complex deposits, composed of HIV peptides and antibodies directed against these antigens, lead to the development of glomerulonephritis [8], consequently leading to tissue immune complexes consisting of HIV antigens, such as p24, gp41, and gp120, bound to IgG or IgA antibodies produced in response to these antigens. [9]

Histopathologic findings of HIV-IgA nephropathy include podocyte hyperplasia and fibrocellular crescents, microcystic tubular dilatation with interstitial inflammatory infiltrate made up of macrophages and lymphocytes with occasional plasma cells, polymorphonuclear leukocytes, and eosinophils, all of these findings related to a greater number of B cells infiltrating the tubule interstitium compared with patients with classic HIVAN [10]

There have been several reports of IgA nephropathy occurring in HIV-infected patients, primarily in Caucasian or Hispanic patients, like our patient who was Hispanic [11]. Two cases of IgA nephropathy in HIV infected patients who were shown to have immune complexes composed of idiotypic IgA antibodies, which were secondary to dysregulation of the immune system associated with HIV infection, a response to anti-HIV antibodies, or continued exposure to HIV-associated peptides [12].

In the study by Eustace et al. prednisone was not associated with an increased risk of infection. However, the use of high-dose corticosteroids in HIV patients was associated with a high risk of avascular necrosis of the femoral head. In the era of modern highly active antiretroviral therapy (HAART), it is unclear what the potential benefits are, if any, of the use of corticosteroids in the treatment of patients with HIV-associated nephropathy (HIVAN), or other HIV-related kidney diseases [13]

Data on therapy for IgA nephropathy in the setting of HIV infection are limited to case reports [14]. Despite limited evidence in HIV infected patients, treatment of patients with IgA nephropathy should include conservative medical therapy with angiotensin-converting-enzyme inhibitors (ACEIs) or angiotensin receptor blockers (ARBs). If the disease is progressive despite optimization of blood pressure control with ACEIs or ARBs, the use of immunosuppressive agents can be considered. More extensive prospective randomized controlled trials are needed to determine the optimal therapy of IgA nephropathy in HIV infected patients [15]. Clinical remission of IgA nephropathy in an HIV-positive patient was reported after combined treatment with tonsillectomy and steroid pulse therapy. The patient continued in remission after 3 years [16].

HSP in adults is usually severe and requires aggressive therapy. Steroids, other immunosuppressive treatment with azathioprine or cyclophosphamide, therapeutic plasma exchange, and more recently rituximab are used without definitive evidence of effectiveness. The treatment of HSP in HIV infected patients is not well codified, and it is based on case reports. The use of ART has been demonstrated to efficiently lead to the disappearance of cutaneous, articular and abdominal signs in some reported cases [15].

## Conclusions

We described the case of a Hispanic male adult with IgA nephropathy and Henoch-Schonlein Purpura in the setting of HIV. The incidence of vasculitis associated with HIV infection is less than 1%. There are few cases reported of IgA nephropathy in the context of HI. Henoch-Schonlein Purpura usually presents in children. We encountered a patient with rare illnesses while in the setting of immunodeficiency.
